# The Roles of Hippo Signaling Transducers Yap and Taz in Chromatin Remodeling

**DOI:** 10.3390/cells8050502

**Published:** 2019-05-24

**Authors:** Ryan E. Hillmer, Brian A. Link

**Affiliations:** Department of Cell Biology, Neurobiology and Anatomy, Medical College of Wisconsin, 8701 Watertown Plank Road, Milwaukee, WI 53226, USA; rhillmer@mcw.edu

**Keywords:** chromatin, epigenetic, transcription, Hippo pathway

## Abstract

Hippo signaling controls cellular processes that ultimately impact organogenesis and homeostasis. Consequently, disease states including cancer can emerge when signaling is deregulated. The major pathway transducers Yap and Taz require cofactors to impart transcriptional control over target genes. Research into Yap/Taz-mediated epigenetic modifications has revealed their association with chromatin-remodeling complex proteins as a means of altering chromatin structure, therefore affecting accessibility and activity of target genes. Specifically, Yap/Taz have been found to associate with factors of the GAGA, Ncoa6, Mediator, Switch/sucrose nonfermentable (SWI/SNF), and Nucleosome Remodeling and Deacetylase (NuRD) chromatin-remodeling complexes to alter the accessibility of target genes. This review highlights the different mechanisms by which Yap/Taz collaborate with other factors to modify DNA packing at specific loci to either activate or repress target gene transcription.

## 1. Introduction

Recruitment and/or activation of transcription factors and proteins capable of altering target gene transcriptional activity is a hallmark of many signaling pathways. Alterations in transcriptional activity can be achieved via recruitment of proteins capable of remodeling the chromatin structure through the modification of nucleosome positioning and histone proteins at regulatory regions of target genes [1,2,3]. Transcriptional activity can also be modulated via direct or indirect recruitment of transcriptional machinery to target gene loci [4]. 

The Hippo signaling pathway governs tissue growth and homeostasis through control over cell proliferation, differentiation, fate, metabolism, and apoptosis [5,6]. Regulation of these cellular processes is ultimately achieved though pathway-mediated localization of the downstream mammalian effectors Yes-associated protein (Yap) and transcriptional activator with PDZ binding motif (Taz/WWTR1), or Yorkie (Yki) in *Drosophila* [7,8]. The pathway itself is comprised of a core kinase cascade involving the subsequent phosphorylation of mammalian STE20-like protein 1/2 (Mst1/2) and large tumor suppressor 1/2 (Lats1/2) kinases. Core kinase activity is bolstered by interaction with Salvador-homolog 1 (Sav1) and MOB kinase activator 1 (Mob1). Mst1/2 interaction with Sav1 reinforces its phosphorylation of Lats1/2, whose phosphorylation activity is enhanced through Mst1/2 phosphorylation of Mob1 [9,10,11]. Sequential phosphorylation events ultimately lead to the phosphorylation of Lats1/2 to induce its interaction with and phosphorylation of downstream pathway effectors Yap and Taz [12,13]. Yap/Taz phosphorylation prevents their nuclear localization and results in cytoplasmic sequestration via binding to the 14-3-3 adaptor protein [14]. Furthermore, targeted degradation of Yap/Taz can be achieved through subsequent phosphorylation by casein kinase 1 [15,16]. Overall activity of the Hippo signaling kinase cascade serves to prevent the transcriptional activity of downstream effectors Yap and Taz. When signaling is not active, Yap/Taz can enter the nucleus and bind to DNA through interaction with cofactors to impart effects on transcription. Canonically, Yap/Taz binding to TEA-domain (TEAD) family members has been shown to induce transcription of target genes [17]. However, direct interaction of Yap/Taz with other DNA-bound cofactors including p73, Tbx5, SMADs, and RUNX1/2 has also been demonstrated [18,19,20,21]. 

Mechanistic studies into how Hippo signaling effectors Yap and Taz influence target gene transcriptional activity has revealed the significance of imparting chromatin alterations at target loci. For example, recent chromatin conformation and transcript expression experiments performed on cardiomyocytes overexpressing YAP suggest a function for YAP in modulating chromatin accessibility [22]. The chromatin landscape of cardiomyocytes expressing a constitutively active form of YAP was found to be in a more accessible conformation at TEAD binding motifs within the genome. Genomic regions characterized by decreased chromatin accessibility with YAP overexpression were also apparent [22]. It is possible, however, that these chromatin changes simply reflect a block in differentiation, as these cells maintain a proliferative, fetal-like state. Transient overexpression of Yap following cellular differentiation will be insightful to discriminate these possibilities. However, bona fide interactions with chromatin-remodeling complexes have been established with the transcriptional output factors of Hippo signaling. Yki/Yap/Taz recruitment of and interaction with chromatin remodelers of the SWI/SNF complex, GAGA factor, Mediator complex, Ncoa6, and NuRD complexes have all been documented as means for Yki/Yap/Taz mediated alterations of target gene transcriptional activity [23,24,25,26,27,28,29,30,31,32,33] and are reviewed in the following sections (Table 1). 

## 2. Interactions of Yki/Yap/Taz with the SWI/SNF Family of ATP-Dependent Chromatin-Remodeling Complexes

The switch/sucrose nonfermentable (SWI/SNF) complex is an ATP-dependent chromatin-remodeling complex first described in yeast and named for the effects of its subunits on altered gene expression related to mating type switching (SWI) and sucrose fermentation (SNF) [35,36]. *Drosophila* SWI/SNF complexes include the Brahma-associated protein complex (BAP) and the Polybromo-containing BAP complex (PBAP), with the Brahma ATPase being a common component of both complexes [37]. Brahma (Brm), Brahma-related gene 1 (Brg1), and associated factors (BAFs) comprise the SWI/SNF complex in vertebrates [38]. Mechanistically, SWI/SNF complex activity is thought to be achieved through chromatin binding and histone positioning mediated by actin-related proteins (Arps), and subsequent DNA-dependent ATPase activity at acetylated histone tails [39,40]. Functionally, SWI/SNF has mainly been implicated in activating gene transcription, although instances of SWI/SNF involvement in gene repression have also been documented [41,42]. Mechanistically, SWI/SNF chromatin-remodeling complexes function to modify nucleosome organization in an ATPase-dependent manner, altering the accessibility of transcription factors to genomic loci. As such, SWI/SNF complexes play important gene regulatory roles in multiple contexts [43,44,45,46]. In relation to Yki/Yap/Taz transcriptional functionality, the Brahma subunit of the SWI/SNF complex has been documented as an important cofactor for transcriptional regulation.

### 2.1. Brahma–Yki Interactions Documented in Drosophila

**Figure 1:**
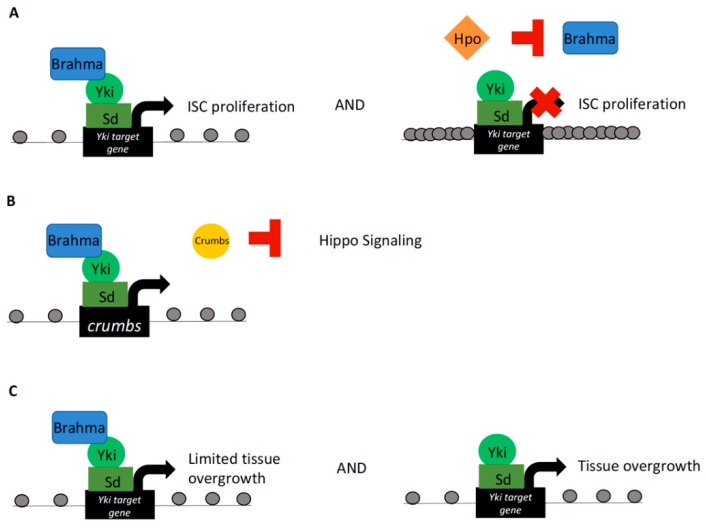
Yorkie interactions with the SWI/SNF complex. (**A**) Brahma and Yki–Sd interact to drive *Drosophila* midgut intestinal stem cell (ISC) proliferation. Notably, cotransfection with Hippo results in caspase-mediated proteolysis of Brahma and loss of complex stability. (**B**) Brahma has also been shown to interact with Yki–Sd at Yki target genes to promote tissue overgrowth in the wing disc and eye. This interaction occurred at the *crumbs* locus, providing evidence for a feed-forward loop of Crumbs-mediated Yki activation in regulation of tissue growth in *Drosophila*. (**C**) In the *Drosophila* wing imaginal disc, *yki* overexpression drives tissue overgrowth. This overgrowth phenotype was exacerbated by *brahma* knockdown, providing evidence for a regulatory role of Brahma in limiting Yki-mediated tissue overgrowth. Spacing of grey dots, representing nucleosomes, represents chromatin compaction.

Analysis of SWI/SNF functionality in *Drosophila* midgut intestinal stem cell (ISC) proliferation and regeneration revealed a requirement of the Brahma subunit in proper regulation of these cellular processes. Known involvement of Yki activity in governing ISC proliferation led to investigations of Brahma–Yki interactions in driving the midgut ISC proliferative capacity [47,48]. Coimmunoprecipitation products of Yki or Scalloped (Sd, the fly homolog of TEAD) subjected to mass spectrometry analyses revealed interactions with multiple BAP complex components, thus suggesting a role of this complex in Yki–Sd-mediated transcriptional activity governing ISC proliferation [29]. Of note, when cotransfected with the Hippo kinase in a cell culture assay, Brahma protein levels were decreased. This reduction in protein levels was found to be induced by Hippo mediated cleavage of Brahma, negatively affecting overall complex stability. Mechanistically, Hippo kinase activity was found to stimulate caspase proteolysis, resulting in cleavage of Brahma. Coincidently, phospho-mediated caspase activation is a known function of the Hippo pathway in *Drosophila* [49]. Furthermore, a cleavage-resistant Brahma mutant was found to promote ISC proliferation [29]. Ultimately, these results suggest that Yki–Sd form a complex with Brahma in the nucleus and that Brahma protein stability is mediated by Hippo kinase activity (Figure 1A). These findings provide support for a regulatory role of Hippo signaling in control of Brahma protein stability and chromatin alterations imparted by BAP SWI/SNF complex recruitment at Yki targets; and subsequent Brahma regulation by the Hippo kinase. 

Other investigations into modulation of the Hippo effector Yki by the SWI/SNF component Brahma in *Drosophila* implicated Brahma–Yki interactions in inducing the transcription of *crumbs*. Crumbs is a large cell junction-associated transmembrane protein known to negatively regulate the Hippo signaling cascade. Knockdown of *brahma* resulted in wing growth reduction and a small eye phenotype, placing Brahma as an integral factor for cell proliferation and overall tissue growth regulation [32]. To genetically test Brahma–Hippo pathway interactions, phenotypes resulting from loss-of-function mutations of expanded or *hippo* coupled with *brahma* knockdown were characterized. Mutations of *expanded* and *hippo* resulted in increased Yki activity and were characterized by tissue overgrowth. This effect was inhibited by *brahma* knockdown. Tissue overgrowth was also observed with direct overexpression of *yki*, and likewise was inhibited by *brahma* knockdown. In addition, Yki–Brahma activity depended on Sd interaction, a similar finding to previous reports [29]. Furthermore, Yki and Brahma were localized to the *crumbs* promoter as shown by chromatin immunoprecipitation (ChIP), therefore implicating Brahma in a feed-forward loop of Crumbs-mediated Yki activation in governing tissue growth (Figure 1B) [32]. Together, these experiments defined Brahma as critical for Yki–Sd function in regards to tissue growth regulation [32]. 

Tissue growth regulation by Brahma–Yki interactions provides the potential for this protein complex to cause cancer and affect tumor growth. Indeed, dysregulation of both SWI/SNF complexes and Hippo pathway activity can result in cancer phenotypes [50,51]. This tumor-promoting activity of Yki is, in certain circumstances, dependent on the activity of the SWI/SNF BAP complex [33]. In the wing imaginal disc, *yki* overexpression coupled with knockdown of *brm* (or other BAP-specific components) was shown to result in wing disc overgrowth, which was exacerbated as compared to *yki* overexpression alone. This demonstrates that BAP can limit *yki*-driven tissue overgrowth. Hyperproliferative wing discs were characteristic of larvae overexpressing Yki or depleted of BAP subunits. Additional malignant features included defects in cell polarity and induction of secreted matrix metalloproteinase 1, which promotes basement membrane degradation [33,52]. Furthermore, depletion of BAP led to ectopic expression of Yki target genes, along with ectopic expression of wing disc growth factors *decapentaplegic* (*dpp*) and Wingless (Wg) [53]. Ectopic expression of *dpp* and Wg was found to augment the tumor-forming phenotype observed in *yki* overexpression/*brm* knockdown wing discs [33]. This research therefore describes a role of the BAP complex as a tumor suppressor in tissues with gain of Yki activity (Figure 1C) and links BAP-mediated chromatin remodeling to cancer phenotypes resulting from dysregulated Hippo signaling. 

### 2.2. BAF-YAP/TAZ Interactions Documented in Mammalian Cells

**Figure 2:**
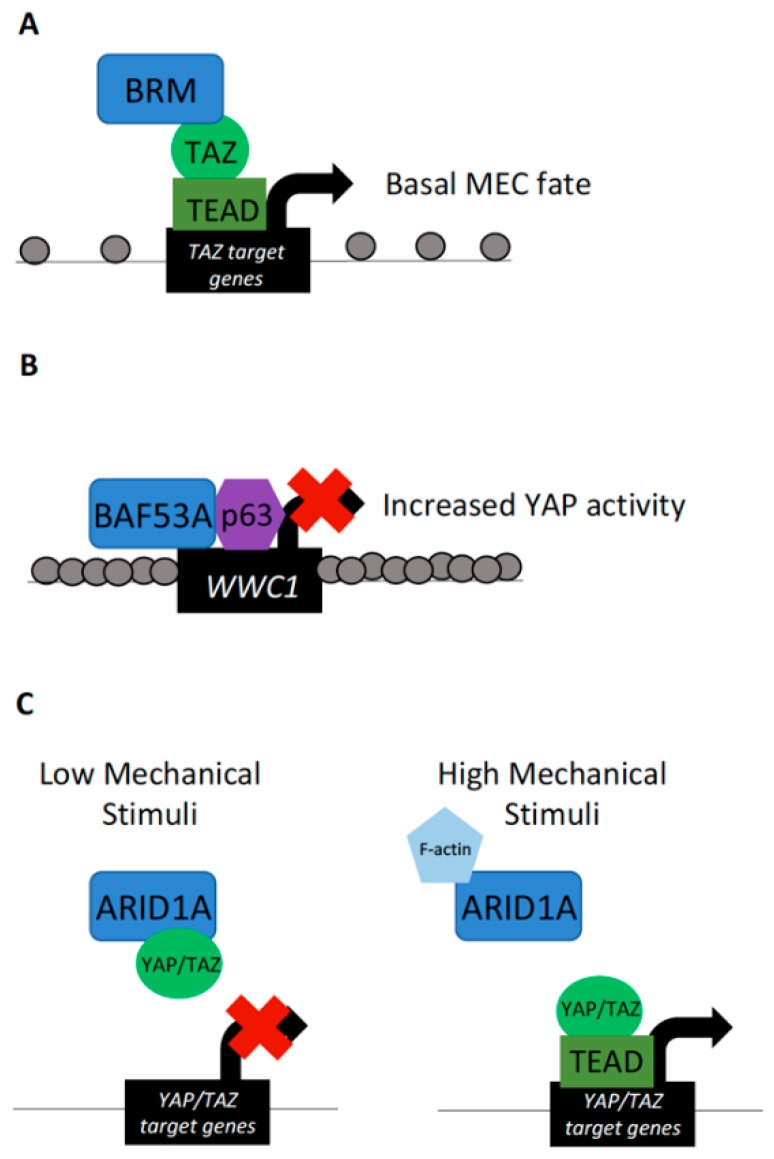
YAP/TAZ interactions with the SWI/SNF complex. (**A**) BRM interaction with TAZ–TEAD at TAZ target loci drives transcription of genes specifying a basal cell fate in mammary epithelial cells (MECs). (**B**) The SWI/SNF subunit Brahma-associated factor 53a (*BAF53A*) interacts with *p63* to inhibit the expression of *WWC1/KIBRA*. This results in the overactivation of YAP and is a hallmark of head and neck squamous cell carcinoma (HNSCC). (**C**) Direct interaction of the SWI/SNF subunit ARID1A with YAP/TAZ under conditions of low mechanical stimuli sequester YAP/TAZ from activating target gene expression. Under conditions of high mechanical stimuli, ARID1A itself is sequestered by nuclear F-actin, allowing YAP/TAZ to bind TEAD at target loci and induce transcription. Of note, interactions of YAP/TAZ with ARID1A does not alter chromatin accessibility at YAP/TAZ target loci. Spacing of grey dots, representing nucleosomes, represents chromatin compaction.

In addition to SWI/SNF–Hippo signaling interactions in *Drosophila*, cooperation is evident in mammalian cells as well. Investigation into human mammary epithelial cell (MEC) lineage switching between basal and luminal cell fates has implicated TAZ as important in controlling this process. Specifically, TAZ depletion in basal cells, where it is localized to the nucleus, results in lineage switching towards a luminal cell fate. Conversely, ectopic nuclear TAZ expression in luminal cells switches their identity to a more basal cell type [24]. To determine how TAZ induces this cell fate switch in MECs, coimmunoprecipitation/mass spectrometry studies were conducted. These experiments revealed interactions between TAZ and the BAF–SWI/SNF complex catalytic subunits BRG1 and BRM. The interaction was dependent on the PPXY motif of BRG1/BRM and the WW domain of TAZ. Of these particular SWI/SNF catalytic subunits, only BRM depletion resulted in a decrease in TAZ target expression and ChIP experiments revealed an enrichment of BRM at genomic regions containing TEAD binding motifs. Furthermore, the TAZ–BRM interaction was necessary for the repression of MECs towards a luminal differentiation fate (Figure 2A), thus providing evidence of TAZ–BRM interactions in mammalian cell lines and importance of this association in governing lineage specification [24]. 

A role for YAP/TAZ working in conjunction with chromatin-remodeling factors may be a more generalized feature of multiple cancers. Dysregulation of the SWI/SNF complex in concert with increased Yki/YAP/TAZ activity has been documented to promote tumor formation in *Drosophila* and mammals [33]. In human cells, transcript levels of the BAF–SWI/SNF subunit *Brahma-associated factor 53a (BAF53A)* has been found to be amplified in head and neck squamous cell carcinoma (HNSCC) along with *p63*. *BAF53A* and *p63*, a DNA-binding transcription factor, form a complex to activate the expression of target genes that promote proliferation and prevent differentiation in HSNCC [31]. These effects on target gene transcription are mediated by *BAF53A-p63* repression of *WWC1* (also known as *KIBRA*), which is a regulator of the Hippo–YAP signaling pathway [54]. Decreased *WWC1* expression was achieved through a *BAF53A*-*p63*-mediated reduction in chromatin accessibility upstream of the *WWC1* transcription start site. Reduced levels of WWC1 subsequently resulted in increased YAP activity (Figure 2B). Furthermore, the finding that the SWI/SNF subunit complexed with *p63* to ultimately activate YAP-mediated transcription in HSCC was correlated with poor survival of HSCC patients [31]. These results lend additional support to the involvement of SWI/SNF complex dysregulation in tumor progression and demonstrate another role of the SWI/SNF chromatin-remodeling complex in mediating Hippo pathway effector activity. 

In contrast to the above-mentioned report of BAF–SWI/SNF in activating TAZ target transcription, this complex has also been shown to directly inhibit YAP and TAZ in cultured mammalian cells. In MCF10AT and HEK293T cells, representing breast and kidney epithelia, YAP/TAZ forms complexes with BAF–SWI/SNF through the ARID1A subunit. Interestingly, regulation of ARID1A–YAP/TAZ is promoted through mechanotransduction, where the interactions of these proteins form under situations in which cells are exposed to low levels of mechanical stimuli [34]. Under situations in which cells are exposed to high levels of mechanical stimuli, ARID1A is sequestered by nuclear F-actin and canonical YAP/TAZ–TEAD complexes form (Figure 2C). In this manner, YAP/TAZ transcriptional activity can be inhibited not only by Hippo signaling activity, but also by interactions with the SWI/SNF complex under differing levels of mechanical stimulation [34]. In this example, BAF–SWI/SNF components also maintain regulatory roles with YAP/TAZ independent of chromatin accessibility regulation. 

## 3. Interactions of *Drosophila* Yki with the Chromatin Protein GAGA Factor and the Mediator Complex

The chromatin protein GAGA factor (GAF) associates with transcriptional machinery components and regulatory protein complexes to alter chromatin structure. GAF is encoded by the *trithorax-like* (*trl*) gene in *Drosophila* and was initially thought to act as a transcriptional activator [55]. GAF has been shown to recruit chromatin-remodeling complexes to induce and maintain nucleosome-free chromatin regions [56]; including PBAP, NURF, and FACT complexes [57,58,59,60]. In addition to involvement in transcriptional activation, GAF has been implicated in transcriptional silencing via interaction with Polycomb group proteins [61,62,63]. Furthermore, in addition to associating with numerous chromatin remodelers, GAF has also been documented to recruit RNA polymerase II [64]. The Mediator complex also associates with transcriptional machinery components, linking the preinitiation complex to RNA polymerase II to recruit transcriptional machinery [4]. The Mediator complex has also been shown to act with p300 to activate chromatin templates, inducing chromatin remodeling and preinitiation complex formation [65,66]. 

**Figure 3:**
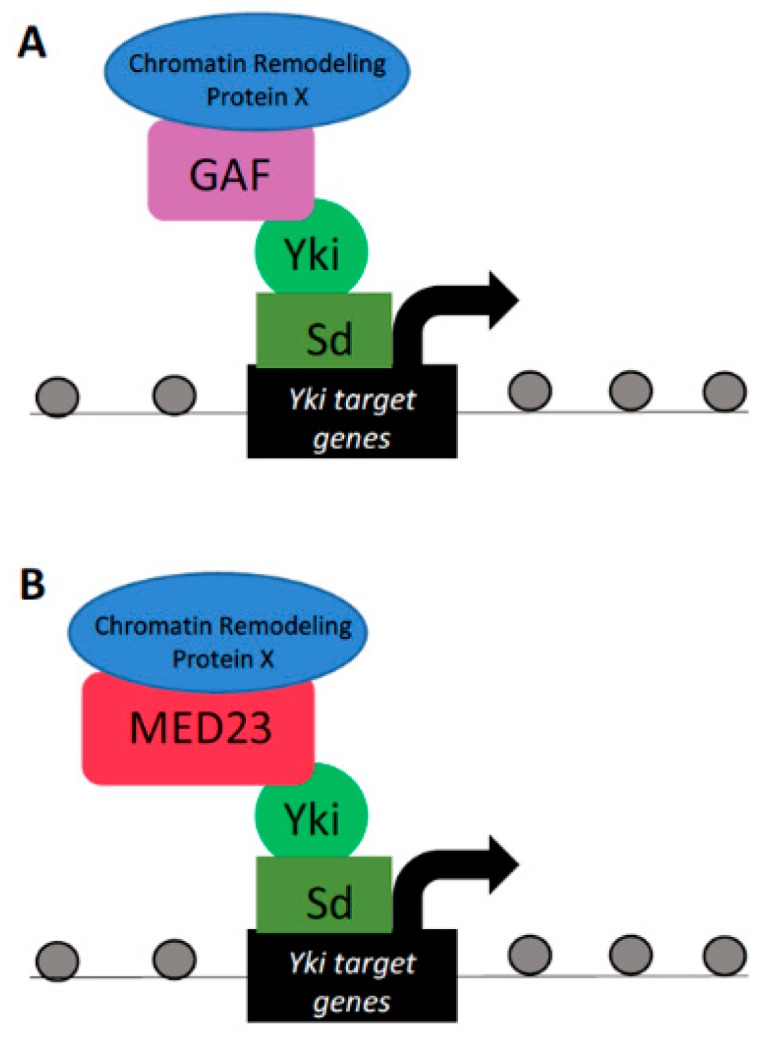
Yorkie interactions with GAF and the Mediator complex. Yki–Sd can interact with the chromatin protein GAF (**A**) or the Mediator complex subunit MED23 (**B**) to drive the transcription of Yki target genes that govern tissue proliferation. Although GAF and the Mediator complex do not contain intrinsic chromatin-remodeling capabilities, they are thought to recruit chromatin-remodeling proteins, yet to be identified, capable of modifying DNA organization.

Although not characterized by intrinsic chromatin-remodeling capabilities itself, GAF acts as a hub for the recruitment of protein complexes with chromatin-remodeling capacities. GAF has been documented to recruit chromatin-remodeling complexes capable of altering chromatin accessibility in a bidirectional manner. In this way, it can either complex with remodelers that induce a nucleosome-free, open chromatin region, or recruit repressor complexes to inhibit chromatin accessibility [57,58,59,60,61,62,63]. In *Drosophila*, GAF has been shown to act as a pertinent cofactor for the Yki/Sd–dE2f1 transcriptional program driving cell proliferation (Figure 3A) [28]. Knockdown of GAF protein in larval wing imaginal discs resulted in reduced cell proliferation. Furthermore, GAF is found at Yki/Sd–dE2f1 target gene promoters, which exhibit reduced expression when GAF is knocked down. The wing discs of larvae in which GAF was knocked down were characterized by slowed proliferation and were smaller in size when compared to control wing discs [28]. These studies in *Drosophila* defined GAF as required for Yki to induce target transcription and ultimately affect cell proliferation in wing progenitor cells. 

Investigation into the chromatin binding of Yki and its interaction with chromatin-remodeling complexes has been studied by ChIP-DNA sequencing (ChIP-seq) characterization [30]. DNA-binding motif analysis of Yki-bound genomic sites revealed an enrichment of *GAGA* sequences. GAF and Yki ChIP-seq peaks were also found to overlap. Furthermore, GAF and Yki proteins bound each other in a manner independent of the Yki WW domain. GAF was also noted as required for the expression of genes that were cobound by Yki and GAF. Consistent with this result, GAF depletion resulted in the reduction of Yki target genes, even under conditions of Yki overexpression [30].

Similar to GAF, the Mediator complex has also been shown to alter DNA packaging via recruitment of chromatin-remodeling complexes [65,66]. The Mediator complex subunit Med23 was shown as a nuclear Yki-associated protein (Figure 3B) [30]. Furthermore, the expression of direct transcriptional targets of Yki was found to be dependent on the Mediator complex, such that downregulation of Mediator decreased Yki target gene expression under conditions of activated Yki [30]. Taken together, these studies provide evidence for Yki recruitment of, and dependency on, chromatin GAF and Mediator complexes in governing Yki-mediated transcriptional regulation over target gene expression. Despite the requirement for the GAF and Mediator proteins in Yki transcriptional output efficiency, the detailed mechanisms by which these complexes modulate chromatin structure at Yki target loci remains elusive and requires further investigation. In addition, studies directed at whether Hippo proteins recruit and precisely regulate factors of the GAF and Mediator complexes are also warranted. 

## 4. Yki/Yap and the Histone Methyltransferase Complex

Posttranslational modifications to histones, including methylation, aid in regulation of transcriptional activity. Histone H3 methylation in particular promotes alterations to chromatin structure that affect transcriptional activity [1]. In this manner, differential methylation of particular lysine residues on histone H3 at different genomic regions promotes transcriptional specificity [67]. The methylation status of histone H3 is regulated by a distinct complex of proteins associated with the Set1 (COMPASS) family of H3K4 histone methyltransferase (HMT) complexes [3,68]. COMPASS H3K4 HMTs include global H3K4 HMT Set1, along with Trithorax (Trx) and Trithorax-related (Trr) histone methyltransferase complexes [68,69]. Trx is primarily dedicated to the regulation of homeotic genes, while Trr is implicated in steroid hormone signaling and H3K4 monomethylation [70]. Of particular importance, the nuclear receptor coactivator 6 (Ncoa6) subunit of the Trr histone methyltransferase complex has been documented to interact with *Drosophila* Yki and mammalian YAP to methylate H3K4 and activate transcription [25,26]. 

**Figure 4:**
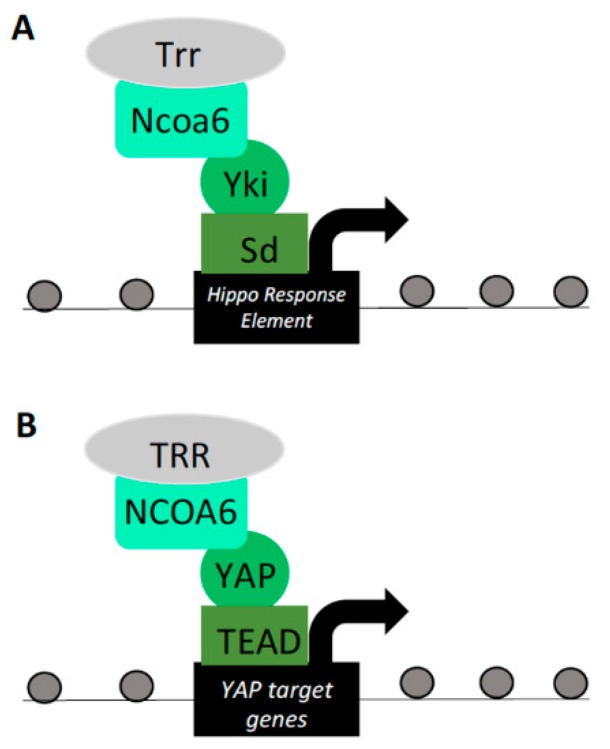
Yorkie/YAP interactions with the histone methyltransferase (HMT) complex. (**A**) Ncoa6, a component of the Trithorax-related (Trr) H3K4 methyltransferase complex, binds to Yki–Sd and is capable of activating a Hippo response element (HRE) reporter in *Drosophila*. Ncoa6 interaction with Yki–Sd drives the expression of Yki target genes, inducing tissue growth and H3K4 methylation at these loci. (**B**) Human NCOA6 interaction with YAP–TEAD drives the expression of YAP target genes, providing evidence for evolutionary conservation between this Yki/YAP–HMT interaction.

Ncoa6, a subunit of the Trithorax-related H3K4 methyltransferase complex, was found to be a positive regulator of the Yki–Sd-driven Hippo-responsive transcriptional reporter in *Drosophila* S2R+ cells [71]. Ncoa6 was also found to harbor PPXY motifs, required for its physical interaction with Yki WW domains [25]. Overexpression of Ncoa6 enhanced Yki–Sd-mediated transcriptional reporter activity and bound the Hippo-responsive DNA elements of the Yki target gene *diap1*. Consistent with a functional role, knockdown of Ncoa6 resulted in decreased expression of *diap1* in adult wings. Furthermore, increased eye size induced by Yki overexpression was ameliorated by knockdown of Ncoa6 [25]. These phenotypes place Ncoa6 as an important player in the regulation of Hippo pathway-mediated target gene expression and subsequent tissue growth. Of note, Ncoa6–Sd was incapable of inducing target transcription in the absence of Yki, suggesting that a complete Ncoa6–Yki–Sd complex is required for Hippo pathway target gene transcription. Furthermore, the H3K4 methylation status of Yki target genes was deleteriously affected by knockdown of either Yki, Ncoa6, or Trr. Therefore, in wing and eye imaginal disc differentiation, Yki activates transcription through the recruitment of the Ncoa6 histone methyltransferase complex (Figure 4A) [25]. 

A similar study investigating Yki recruitment of a histone methyl transferase to induce target gene transcription also found that ChIP-seq peaks for Yki overlapped with peaks for the H3K4me3 histone modification. Furthermore, targeting Yki to a novel chromosomal locus induced H3K4me3 modifications in a WW domain-dependent manner [26]. Yki was similarly found to bind to Ncoa6 in cultured *Drosophila* S2 cells and Ncoa6 and Trr were both shown to interact with Yki. Binding of Trr on genomic DNA overlaps with Yki-bound regions, suggesting co-occupancy at Yki target genes. As reported in a similar study, Ncoa6 recruitment was sufficient to drive Yki transcriptional activity [25,26] and was required for Yki-mediated eye-overgrowth phenotypes. Concerning the phylogenetic extension of this Ncoa6–Yki interaction, human YAP and NCOA6 were found to bind in a PPXY and WW domain-dependent manner (Figure 4B). Reduction of NCOA6 decreased transcriptional activation of YAP targets [26]. This observed NCOA6–YAP association in mammalian cells supports an evolutionarily conserved mechanism by which YAP recruits histone methyltransferases to modulate chromatin structure and regulate target gene transcription. 

## 5. Interactions of YAP/TAZ/TEAD with the NuRD Complex

While the cases documented above describe how Hippo signaling components collaborate with chromatin-remodeling factors to increase gene expression, alterations of chromatin structure to decrease underlying gene activity are also critical for proper genome regulation. One such method of decreasing chromatin accessibility and transcriptional activity is through recruitment of the repressive nucleosome-remodeling and deacetylase (NuRD) complex [72]. The NuRD complex is distinct in that it couples both ATP-dependent chromatin remodeling with histone deacetylase activity as a means of repressing transcriptional activity [73]. In this manner, NuRD induces the compaction of nucleosomes around bound regulatory regions to restrict genomic accessibility. The main ATP-dependent chromatin-remodeling NuRD components are chromodomain helicase DNA-binding proteins 3/4 (CHD3/4), while histone deacetylase 1/2 (HDAC1/2) activity is responsible for the removal of methyl groups from lysine residues at bound genomic loci [72,73]. Relevant to Hippo signaling, human YAP/TAZ/TEAD have been shown to interact with the NuRD complex as a means of repressing target gene activity [23,27]. Although the NuRD complex can activate target transcription in some circumstances, documented instances of interactions with YAP/TAZ/TEAD are all repressive in nature [74].

A regulatory complex comprised of Hippo effectors YAP/TAZ–TEAD, TGF-β effectors SMAD2/3, and the pluripotency regulator OCT4, termed the TEAD–SMAD–OCT4 (TSO) complex, has been implicated in governing the switch of human embryonic stem cells (hESCs) to either maintain their pluripotency status or specify their fate towards a mesendoderm (ME) cell type. Cell fate is regulated in part through differential interactions of the NuRD repressor complex to first inhibit expression of loci involved in ME specification until fate induction is triggered, at which point the TSO complex is remodeled to allow for gene activation [23]. The interaction of TSO and NuRD was ameliorated by YAP/TAZ knockdown, suggesting that YAP/TAZ are the main proteins in TSO responsible for the functional assembly of NuRD. Upon addition of ME fate-driving factors to the culture media, NuRD was replaced by FOXH1 to facilitate activation of the ME target loci [23]. This study was the first to document a role for YAP/TAZ–TEAD in NuRD complex activity to repress target transcription.

**Figure 5:**
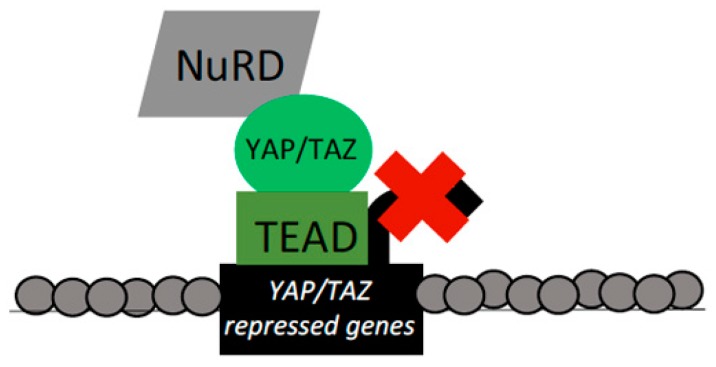
YAP/TAZ/TEAD interactions with the NuRD complex. YAP/TAZ–TEAD bind targets and recruit the NuRD complex to repress target expression. This repression is mediated through dual ATP-dependent chromatin remodeling and histone deacetylase (HDAC)-mediated histone deacetylase functions of the NuRD complex to ultimately reduce chromatin accessibility. YAP/TAZ targets repressed by NuRD recruitment included genes that drive apoptosis and promote senescence.

YAP/TAZ–TEAD collaboration with the NuRD complex to repress target gene transcription has also been verified in recent studies. A screen for genes directly regulated by *YAP* overexpression revealed transcripts that both increased and decreased under these conditions. This represents another example where YAP/TAZ, which have canonically been thought of as transcriptional activators, are capable of repressing target gene expression. This transcriptional corepressive role of YAP/TAZ was found to depend on TEAD binding, and YAP/TAZ–TEAD were found to directly bind repressed target loci [27]. The observed repression of YAP/TAZ targets was shown to result from alterations in chromatin at repressed targets, as indicated by effects on histone acetylation and factor occupancy at target promoter regions. This alteration in DNA organization of loci repressed by YAP/TAZ was mediated by recruitment of the NuRD complex, which bound repressed YAP/TAZ targets in a TEAD-dependent manner (Figure 5). Of note, the genes repressed by YAP/TAZ–TEAD–NuRD included those that encode proteins that promote senescence or drive apoptosis [27]. These results document the capacity of YAP/TAZ as transcriptional corepressors and define a mechanism in which YAP/TAZ–TEAD recruit and/or activate the NuRD complex at regulatory regions of target genes to repress their expression and allow for cell proliferation and survival.

## 6. Summary and Future Directions 

The Hippo signaling pathway has been documented to control tissue growth and homeostasis through its regulation of downstream effectors Yki/YAP and TAZ [5,7]. Canonically, Yki/YAP/TAZ have been described as transcriptional coactivators, and investigations into the mechanisms behind this coactivator role have revealed the importance of direct partnership with chromatin-modifying protein complexes. Specifically, Yki/YAP/TAZ have been shown to interact with chromatin-remodeling complexes of the SWI/SNF family, GAGA factor, Mediator complex, and multiple histone methyltransferases to aid in their activation of target gene transcription through modification of DNA packing and organization [24,25,26,28,29,30,31,32,33,34]. More recently, a transcriptional repressive role of YAP/TAZ/TEAD has also been described, which is mediated through recruitment of the NuRD complex [23,27]. Therefore, effects of Yki/YAP/TAZ on target gene activity are context-specific and highly dependent on the interaction with protein complexes capable of remodeling nucleosome positioning in an ATP-dependent manner and imparting posttranslational modifications to histones [1,41]. 

One area of needed investigation is to better define the mechanisms that underlie the specificity of interactions between Hippo components and chromatin-remodeling complexes. *Drosophila* studies of Hippo and Brahma complexes highlight some of the complexity. Investigations into Yki recruitment of BAP–SWI/SNF chromatin-remodeling complexes demonstrated Yki–Sd interaction with the BAP–SWI/SNF subunit Brahma and regulation of Brahma protein stability by the Hippo kinase [29]. Brahma–Yki interactions were also shown to induce the expression of Yki target genes and influence tissue overgrowth [32]. Interestingly, different SWI/SNF subunits have been shown to be either augmentative or restrictive to Yki-mediated tissue overgrowth, indicating context specificity into the differential regulation of SWI/SNF on Yki activity [32,33]. More focused studies to uncover the basis of differential interactions are warranted, as are studies to address diverse roles of YAP/TAZ–SWI/SNF interactions in vertebrates. 

Of relevance to this issue, recent work has demonstrated a role of the activator protein 1 (AP-1) in driving YAP/TAZ/TEAD activity at distal enhancers [75]. Activated target genes govern the onset of the S phase and cell mitosis, providing for a means of promoting tumorigenesis [75]. Furthermore, evidence of YAP–TEAD driving the expression of AP-1 targets has been shown to foster tumor growth [76], and AP-1 has been shown to co-occupy enhancer and promoter regions along with Tead4 in multiple cancer cell types [77]. AP-1 has also been documented to play a pertinent role in linking YAP/TAZ activity with regulation of the TGF-β/Smad3 signaling axis through promoting expression of Smad7 [78]. Interestingly, AP-1 has the capacity to recruit the BAF–SWI/SNF chromatin-remodeling complex to alter chromatin accessibility at enhancers [79]. These documented instances of YAP/TAZ/TEAD interaction with AP-1 to drive target gene expression provides for yet another avenue in which YAP/TAZ may work in conjunction with SWI/SNF chromatin-remodeling complexes to remodel and regulate target genes. Further investigation is needed to explore this possibility. 

Interactions of Yki with the GAGA factor and the Mediator complex provide a coactivator function in facilitating Yki target transcription [28,30]. Despite the general conclusion that Yki and GAGA or Mediator protein binding are important in regulating target gene transcription, the underlying chromatin-remodeling mechanisms and protein complexes that mediate DNA reorganization remain unknown. As GAGA factor and the Mediator complex can associate with different chromatin-remodeling factors [57,58,59,60,61,62,63,65,66], investigations are needed to identify which specific complexes are involved and under what conditions they bind. 

Gene repressive activity by YAP/TAZ has been shown to depend on NuRD complex recruitment. The NuRD complex, which has both nucleosome-remodeling and histone deacetylase activity, can be recruited by YAP/TAZ–TEAD to render chromatin inaccessible [23,27]. The interaction of YAP/TAZ with NuRD is commonly dependent on TEAD binding [27,73]. However, YAP/TAZ interactions with other DNA-bound factors provide the potential for NuRD recruitment under TEAD-independent contexts [18,19,20,21]. In support of this possibility, Yap overexpression in adult cardiomyocytes drives chromatin remodeling, promoting both open and closed states. While many of these remodeled regions showed TEAD consensus sites, loci that underwent compaction were not enriched for TEADs [22]. Future studies are needed to explore the role of non-TEAD partners of YAP/TAZ that may augment NuRD or other chromatin-modulating complexes to repress gene expression. 

Additional questions regarding Hippo signaling and chromatin remodeling center on the relative timing of interactions between YAP/TAZ–TEAD (or other partners), chromatin-remodeling complexes, and DNA. Do chromatin-remodeling complexes recruit YAP/TAZ–TEAD/other to DNA or vice versa? Is enzymatic activity or target specificity altered by these interactions? What cell states facilitate the protein interactions? Importantly, can the relationships between Hippo components and chromatin remodeling be targeted for therapeutic purposes, particularly with regard to cancer and tissue repair following injury? Cumulatively, the documented collaborations of Yki/YAP/TAZ with chromatin-remodeling factors reviewed herein provides insight into the diverse mechanisms by which Hippo signaling controls gene regulation. With ongoing investigations probing cell-type and tissue-specific roles of the Hippo pathway in governing transcription, it is likely that additional interactions between Yki/YAP/TAZ and the machinery that manages chromatin structure will be discovered. 

## Figures and Tables

**Table 1:** Documented Yki/YAP/TAZ interactions with chromatin-modifying proteins.

Interacting Chromatin Modifying Protein or Complex	Conclusion	Reference(s)
SWI/SNF complex (direct)	Brahma–Yki/Sd interact in the nucleus.	[29,30,32]
	Brahma–Yki/Sd bind to Yki targets.	[30,32]
	Knockdown of Brahma inhibits Yki-mediated tissue overgrowth.	[32]
	Knockdown of Brahma exacerbates Yki-mediated tissue overgrowth.	[33]
	BAP knockdown increases Yki target expression.	[33]
	TAZ/BRM interact via PPXY-WW domains to increase TAZ target expression.	[24]
SWI/SNF complex (indirect)	*ACTL6A*-*p63* inhibits *KIBRA* expression to increase YAP activity.	[31]
	ARID1A sequesters YAP/TAZ from binding to TEAD to decrease YAP activity.	[34]
GAGA factor (direct)	GAF–Yki/dE2f1 bind to Yki targets, increasing their expression and overall cell proliferation.	[28]
	GAF ChIP-seq peaks overlap with Yki ChIP-seq peaks.	[30]
	Yki–GAF interactions occur in a WW domain-independent manner.	[30]
Mediator complex (direct)	Mediator–Yki interact in the nucleus and increase Yki target transcription.	[30]
Histone methyltransferase complex (direct)	Ncoa6–Yki/Sd interact via PPXY-WW domains at Yki targets to drive transcription.	[25,26]
	NCOA6–YAP interact and increase YAP target gene transcription.	[26]
NuRD complex (direct)	YAP/TAZ/TEAD–NuRD interact within the TSO complex to buffer/inhibit expression of pluripotency/ME specification genes.	[23]
	YAP/TAZ/TEAD–NuRD interact to epigenetically repress target gene activity to promote proliferation.	[27]

Note: For full names of gene symbols, see Abbreviations list at the end of this article.

## Abbreviations

HUGO gene nomenclature for gene names and symbols was used within the text.

**Abbreviation**	**Full Name**
ARIDA	AT-Rich Interactive Domain-Containing Protein 1A
Arp	Actin-related protein
BAF	Brm/Brg1-Associated Factor
BAF53a	Brahma-Associated Factor 53a
BAP	Brahma-Associated Protein Complex
Brg1/BRG1	Brahma-Related Gene 1
Brm/BRM	Brahma
CHD3/4	Chromodomain Helicase DNA-Binding Protein 3/4
ChIP	Chromatin Immunoprecipitation
Co-IP	Coimmunoprecipitation
COMPASS	Complex of Proteins Associated with Set1
Crb	Crumbs
dE2f1	E2f Transcription Factor 1
diap1	death-associated inhibitor of apoptosis 1
dpp	decapentaplegic
FACT	Facilitates Chromatin Transcription Complex
FOXH1	Forkhead Box H1
GAF	GAGA factor
HDAC1/2	Histone Deacetylase 1/2
hESCs	Human Embryonic Stem Cells
HMT	Histone Methyltransferase
HNSCC	Head and Neck Squamous Cell Carcinoma
HRE	Hippo Response Element
ISC	Intestinal Stem Cell
Lats1/2	Large Tumor Suppressor Kinase 1/2
ME	Mesendoderm
MEC	Mammary Epithelial Cell
Med23	Mediator Complex Subunit 23
Mob1	MOB Kinase Activator 1
MS	Mass Spectrometry
Mst1/2	Mammalian STE20-like Kinase 1/2
Ncoa6	Nuclear Receptor Co-Activator 6
NuRD	Nucleosome-Remodeling and Deacetylase Complex
NURF	Nucleosome-Remodeling Factor Complex
OCT4	Octamer-Binding Transcription Factor 4
PBAP	Polybromo-Containing BAP Complex
RUNX1/2	Runt-Related Transcription Factor 1/2
Sav1	Salvador-Homolog 1
Sd	Scalloped
SMAD	*C. elegans* “Small” Worm Phenotype *Drosophila* Mothers Against Decapentaplegic
SWI/SNF	Switch/Sucrose Nonfermentable Complex
Taz	Transcriptional Coactivator with PDZ-Binding Motif
Tbx5	T-Box Transcription Factor 5
TEAD	TEA-Domain
TGF-β	Transforming Growth Factor β
Trl	Trithorax-like
Trr	Trithorax-related
Trx	Trithorax
TSO	TEAD–SMAD–OCT4 Complex
Wg	Wingless
WWC1/ KIBRA	WW Domain-Containing Protein 1 Kidney and Brain Expressed Protein
Yap	Yes-Associated Protein
Yki	Yorkie
